# Characteristics of equine summer eczema with emphasis on differences between Finnhorses and Icelandic horses in a 11-year study

**DOI:** 10.1186/1751-0147-51-29

**Published:** 2009-07-14

**Authors:** Raija E Hallamaa

**Affiliations:** 1 Veterinary Clinic, Pisteenkaari 4, 03100 Nummela, Finland

## Abstract

Summer eczema, allergic dermatitis of the horse, was studied on 275 affected horses in Finland in 1997–2007. Features of the horses, clinical signs of the disease and owners' opinions of aggravating factors were recorded. Differences, especially, between two of the native Scandinavian horse breeds, the Finnhorse and the Icelandic horse, were evaluated. The study was based on clinical examination and information from the owners. Of the horses, 50% were Finnhorses, 26% Icelandic horses and 24% consisted of different breeds of ponies and other horses. Of the Finnhorses, 76% had summer eczema by the age of 5 years, but in the Icelandic horses born in Finland the average age at onset was 7 years. The vast majority of the horses, 75%, had moderate clinical signs, while 16% showed severe and 9% mild. The severity of clinical signs did not depend on the duration of the disease nor was it related to the age at onset. The only linkage to severity was the breed of the horse or import from Iceland; New Forest ponies and imported Icelandic horses showed severe clinical signs significantly more often than Finnhorses. Of the owners, 38% regarded insects as the only aggravating factor, 24% mentioned several simultaneous factors, including grass fodder and sunlight, while 22% could not specify any. In Finland, a typical horse breed suffering from summer eczema is the Finnhorse and the characteristics of the disease are mainly uniform with the other breeds affected. Equine summer eczema seems to be aggravated by various combinations of environmental factors.

## Introduction

**Figure 1:**
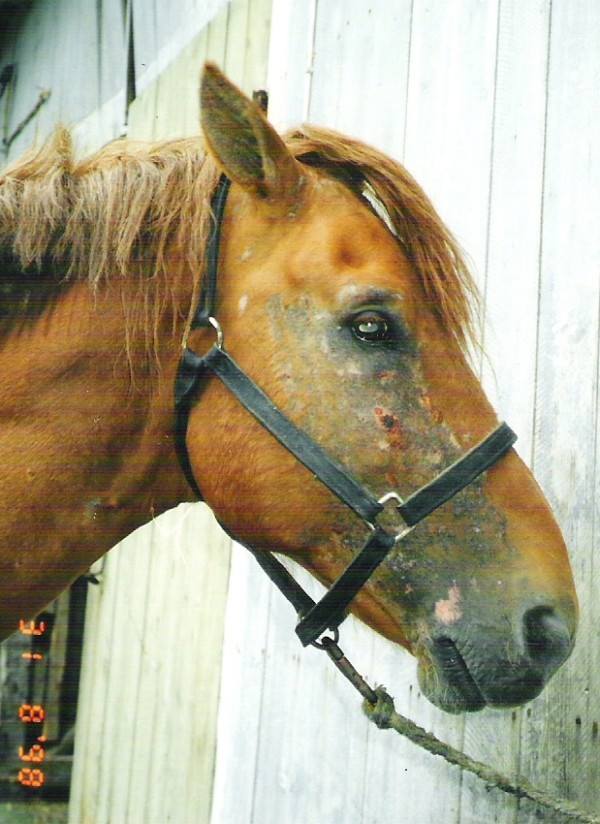
**Finnhorse with severe clinical signs of summer eczema**.

Summer eczema (Queensland itch, sweet itch) is a seasonally recurring allergic skin disease of the horse. It is the most common allergic skin disease and one of the commonest dermatologic diagnoses in the horse [1,2]. The aetiology is usually associated with biting insects, especially species of *Culicoides *[1,3], and therefore the disease is also called insect hypersensitivity or insect bite hypersensitivity, IBH [4,5]. A typical clinical sign is pruritus with following skin lesions and secondary infections (Fig. 1). The mane and tail are most commonly affected, however, horses with severe signs may have lesions on large areas of the entire body [6,7]. A diagnosis is based on clinical examination and typical, seasonally occurring signs [2,5,8,9]. Differential diagnoses are parasitic skin diseases, dermatophytosis and other allergic diseases [1]. Summer eczema is common in Icelandic horses which are exported and thus have not been exposed to bites of insects before import [6-8,10]. The main aim of treatment is to minimize contact with insects and relieve allergic symptoms. Insecticides, special blankets, antihistamines, glucocorticoids or an autogenous serum preparation have been used [1,2,11]. However, treatment is challenging.

The aim of this study was to summarize the features of equine summer eczema in a long-standing study, especially on the Finnhorse, the native Finnish horse breed, and the Icelandic horse and to compare the findings, also with other breeds affected. Additionally, owners' opinions of aggravating factors were delineated.

## Materials and methods

In total, 275 horses suffering from typical, seasonally occuring signs of equine summer eczema entered this study during 1997–2007. Horses were from different regions of Finland and the annual number of new patients varied from 11 to 45. The study consisted of horses whose owners or local veterinarians contacted the author due to summer eczema. The diagnosis was based on a clinical examination by the author or by local veterinarians. Only horses which had clinical signs during thermal summer (the mean temperature of day above +10°C) were included, while horses with pruritic signs before or after this summer season were excluded.

### Data of the horses and the disease

Information on the horses, including breed, age, sex, country of birth and time of import to Finland, was acquired by questionnaire and interviews. In addition, features of the disease were recorded: the age at outbreak, duration of the disease and severity of clinical signs. Owners' opinions concerning aggravating factors were also recorded without using any proposed list of alternatives. The clinical signs were categorised by the author as mild, moderate or severe. Signs were regarded as mild, if pruritus in the mane, tail or body, was the only symptom. Pruritus with mild skin affection in the mane, tail and/or body, was classified as moderate signs, while horses with pruritus and large regional skin lesions in the mane, tail and body were graded as having severe clinical signs (Fig. 1). In addition, the first and last contact of the owners relating to symptoms of summer eczema and the exceptionally cold, rainy or dry periods in each summer season were recorded by the author. Full data of all the horses was not possible to get, especially, when a horse had had many previous owners. Hence the numbers of horses vary in different variables and are then presented in parentheses.

### Statistical analyses

The differences between the qualitative variables with non-parametric distribution were evaluated by Fisher's exact test. The *t *test was used when the differences between the quantitative variables with parametric distribution were analysed. One way analysis of variance was used, when the relation between the age of outbreak or duration of disease and severity of clinical signs was assessed. Two sided *P*-values < 0.05 were considered significant. In all tests, StatsDirect statistical software program (StatsDirect Ltd, Sale, Cheshire, UK) was used.

## Results

The 275 horses which entered the study consisted of 139 Finnhorses, 71 Icelandic horses, 45 ponies, 12 horses of different breeds and 8 horses whose breed was not known (Table 1). All 139 Finnhorses were born in Finland. Of the Icelandic horses, 56 were born in Iceland, 11 in Finland, while the origin of 4 horses remained unknown. In the group of ponies, Shetland and New Forest ponies were the commonest breeds, both of them represented 33% of the affected ponies, while all the rest were of different pony breeds (Table 1).

**Table 1:** Breed distribution, age at onset and duration of disease in 275 horses suffering from summer eczema in Finland.

Breeds	Number of horses		Age at onset	Duration of disease
			mean ± sd/years	mean ± sd/years
Finnhorse	139	(50.5%)	4.6 ± 3.6	3.6 ± 2.7
Icelandic horse	71	(25.8%)	9.0 ± 2.9	3.0 ± 1.9
Shetland pony	15	(5.5%)	3.8 ± 3.1	3.5 ± 1.2
New Forest pony	15	(5.5%)	4.5 ± 3.6	3.7 ± 2.5
Trakehner	3	(1.1%)		
Finnish warmblood	3	(1.1%)		
Russ pony	3	(1.1%)		
Welsh Mountain pony	2	(0.7%)		
Andalusian	1	(0.4%)		
Cob	1	(0.4%)		
Friesian	1	(0.4%)		
Huzu pony	1	(0.4%)		
Welsh pony	1	(0.4%)		
Pony, undefined breed	8	(2.9%)		
Warmblood horse, undefined	3	(1.1%)		
				
Breed not known	8			

Total	275			

Age at onset and duration of disease are listed in the commonest breeds affected.

**Figure 2:**
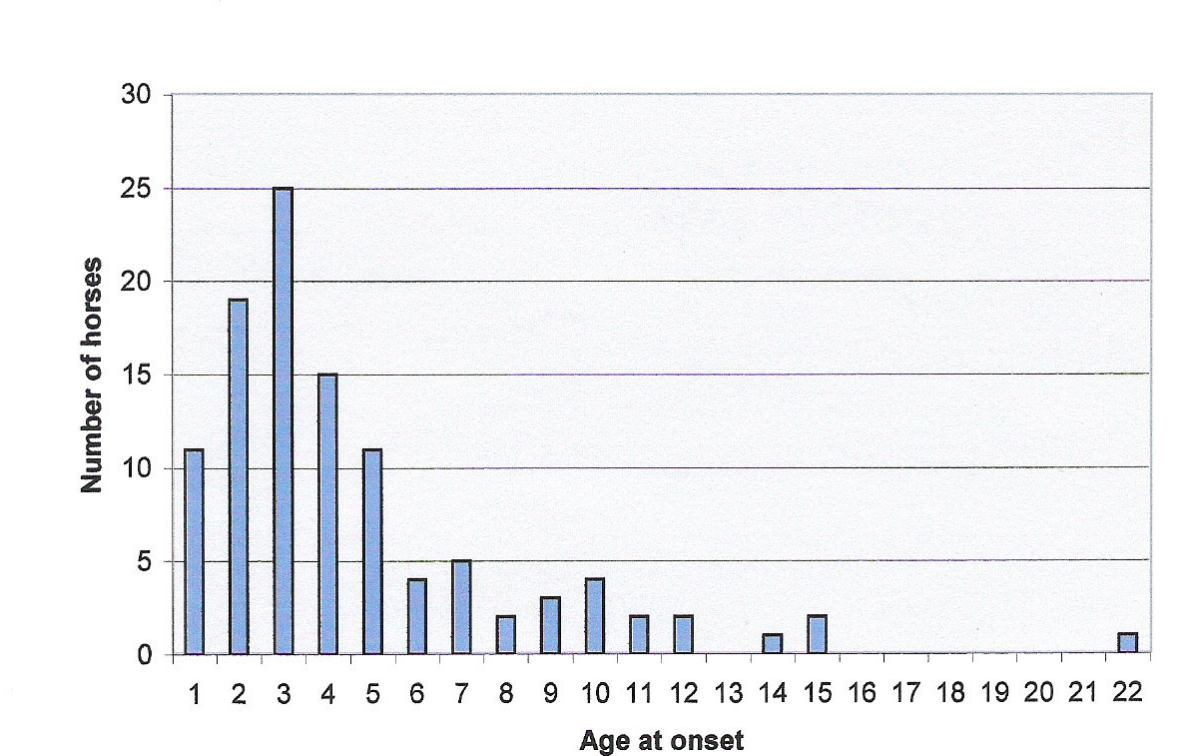
**Age at onset of summer eczema in 107 Finnhorses**.

Of the horses, 48% were female and 52% male; geldings and stallions formed 82% and 18%, respectively. The mean age of the horses was 9.3 ± 4.5 years (range 1–25). The average age at onset was 6.1 ± 4.1 years (range 0.5–22, n = 202). The detailed information on the age at onset or the duration of the disease in the commonest breeds affected is presented in Table 1 and illustrated in Finnhorses in Figure 2. In the Icelandic horses born in Finland, the mean age at onset was 6.9 ± 4.4 years (range 2–15) and, however, did not significantly differ from the age of the Finnhorses (*P *= 0.0611). Of the imported Icelandic horses, 74% developed summer eczema by the third summer after export (mean 3.0 ± 2.2 summers, range 1–7). When the horses entered this study, they had suffered from summer eczema for approximately 3.3 ± 2.4 years (range 1–13 years, n = 217, Table 1). Of the horses, 81% had had symptoms during two consecutive summers or more.

The severity of clinical signs varied from mild to severe; 9% of the horses had mild, 75% moderate and 16% severe signs. Horses with mild clinical signs had suffered from the disease for approximately 2.6 ± 1.8 years, horses with moderate signs 3.5 ± 2.5 years and horses with severe signs 2.8 ± 1.8 years. No significant association was found between the duration and the severity of the disease. The mean age of the outbreak in the horses with mild signs was 5.4 ± 3.9 years, with moderate 6.2 ± 4.0 and with severe 6.3 ± 4.5. In the Finnhorses, the respective values were 4.7 ± 3.6, 4.6 ± 3.6 and 4.1 ± 4.2 years. The age at outbreak did not show significant relation to the severity of clinical signs; neither in the whole group of horses nor in the Finnhorses. No significant difference in the severity of signs could be found between male and female horses. The severity of clinical signs in the most commonly affected breeds in the study is presented in Table 2. Icelandic horses showed significantly more often severe clinical signs than Finnhorses (*P *= 0.0295). When imported Icelandic horses and Icelandic horses born in Finland were evaluated separately, the former group had significantly more commonly (*P *= 0.0183) severe signs than the Finnhorses, whereas the latter group did not (*P *= 0.6176). No significant difference was found in the severity of the disease between imported Icelandic horses and Icelandic horses born in Finland. An exceptionally high proportion of the New Forest ponies showed severe clinical signs (Table 2); significantly more than of the Finnhorses (*P *= 0.0082). Of the 15 New Forest ponies, 12 were born in Finland and the difference was also significant (*P *= 0.0186) between these ponies and Finnhorses. However, New Forest ponies did not show significantly more often severe signs as compared with Icelandic horses, whether or not imported. The severity of the disease in Shetland ponies did not differ from that of the Finnhorses.

**Table 2:** Seriousness of summer eczema in the most commonly affected horse breeds in Finland.

Breed	Seriousness of clinical signs			
	Mild	moderate	severe	Number of horses
Finnhorse	17 (14%)	95 (75%)	14 (11%)	126
Icelandic horse, born in Finland	0 (0%)	9 (82%)	2 (18%)	11
Icelandic horse, imported	2 (4%)	34 (69%)	13 (27%)	49
Shetland pony	0 (0%)	9 (90%)	1 (10%)	10
New Forest pony	0 (0%)	6 (55%)	5 (45%)	11

Total	19 (9%)	153 (74%)	35 (17%)	207

Typically, most of the horses showed clinical signs from May to October. However, the time of onset varied; some of the horses had signs already from May, while others showed no signs until in the late summer. The mean period when owners annually took contact due to summer eczema was 147 ± 15 day, *i.e*. approximately five months. All the horses had been exposed to sunlight and biting insects and most of the horses had been kept on pasture. Of the owners (n = 241), 64% regarded biting insects as one of the aggravating factors, 19% mentioned grass fodder and 12% sunlight, while 22% of the owners could not specify any single factor. Insects were regarded as the only aggravating factor by 38% of the owners, grass fodder 5% and sunlight 2%. Of the owners, 24% mentioned several aggravating factors. Regarding grass fodder, some of the owners mentioned that the species *Trifolia *or heavily used fertilizers seemed to aggravate clinical signs. When the weather conditions were evaluated, 15% of the owners answered that signs were aggravated in sunny or hot and humid weather. No other weather condition was mentioned to have marked influence on aggravation.

## Discussion

The vast majority of the affected horses were Finnhorses. The Finnhorse is a native Finnish coldblood horse breed and it has been officially bred in Finland for 100 years. During the 11-year period of the study, the average number of Finnhorses in Finland was 19 500 and the number of Icelandic horses 1 300. Thus the disease seemed to be more usual among Icelandic horses than among Finnhorses in the present study. However, summer eczema seemed not to be more typical in Icelandic horses born in Finland than in Finnhorses. An exceptionally small number of the affected horses were warmblood, although the total number of warmblood horses in Finland have continuously been about 10 000 greater than the number of Finnhorses during the period of this study. However, insect hypersensitivity has been commonly found in warmblood horses, especially in Arab horses and thoroughbreds [4,9].

Female and male horses were almost equally affected, as has been observed in earlier studies [6,9-11]. The average age at onset was about six years, however, when the age was assessed separately in the Finnhorses and Icelandic horses born in Finland, the latter group tended to get symptoms about two years later than Finnhorses. The explanation for this remained unresolved. Imported Icelandic horses developed clinical signs mainly by the third summer season, as was shown in earlier studies in Sweden and other European countries [7,10], but rarely as late as four years after import, as found to be an average time in Norway [6]. In the present study, Icelandic horses born in Finland became affected about one year or more later than in the previous studies in Norway [6] or in Sweden [10]. Both of these previous studies [6,10] showed that Icelandic-born horses, following exposure, tended to get the disease earlier than those not born in Iceland, as was also found in the present study.

The severity of clinical signs could not be related to the duration of the disease nor to the age at onset. In the present study, the initial signs seem not to be aggravated by years as tended to be the case in the previous study with a smaller number of horses [10]. Additionally, Icelandic horses born in Sweden developed moderate to severe clinical signs at a significantly younger age than horses with mild signs [10]. However, no significant difference between the ages of Finnhorses with mild to severe signs could be found in the present study. The severity of clinical signs *per se *may be difficult to assess between different studies, since the scale of the signs may vary. The categorization of signs has to be made as clear as possible, especially when the variable is qualitative and the study is based primarily on questionnaires. In the present study, the small number of horses with mild or severe signs may be partly due to the strict limits in the evaluation of clinical signs. Additionally, owners probably do not contact a veterinarian, if a horse has only mild signs such as pruritus without skin lesions.

Both Icelandic horses and New Forest ponies had severe clinical signs significantly more often than Finnhorses. However, no difference was found between the Icelandic horses born in Iceland or Finland, unlike in the earlier study with a greater number of Icelandic horses [10]. It has been suggested that Icelandic horses exposed to biting insects as adults will more commonly develop allergic dermatitis than the horses exposed since birth [7,10]. This was also supported by the present study, since the disease seemed to be more prevalent in imported Icelandic horses than in Finnhorses or Icelandic horses born in Finland.

In Israel, *Culicoides *hypersensitivity has been found to be rare on farms being situated on highlands, 800 m above sea level, which should be noted in epidemiological studies [9]. However, the altitude was not a confounding factor in the present study, since virtually all land where horses are kept in Finland are below the level of 800 m. In the recent study [5], it was shown that climate and habitat factors affect the prevalence of IBH; the disease was less common in areas with rainy and cold days or sandy soil with scanty vegetation. In the present study, information about climate or habitat was not actively collected; however, exceptional weather conditions and the owners' opinions of aggravating factors were registered in each year. According to these notes, 15% of the owners regarded sunny or hot and humid days as more difficult for the horses than other weather conditions. Similarly, the suddenly cold days in early summer seemed to decrease the contact by the owners.

The term – summer eczema – probably best describes the entity of this allergic skin disease, especially in Scandinavian countries. The summer time, thermal summer, usually begins in Finland in May and lasts to October (from June to September in northern part of Finland) and the contacts of owners due to clinical signs of summer eczema were clearly restricted to this period. In the summer season in the northern countries, horses are exposed to at least three factors that are not extant in winter: sunlight, pasture fodder and insects. In the previous study by Jose-Cunilleras et al. [12], most of the horses with allergic dermatitis reacted more likely, not only to insects, but also to various allergens, including tree and grass allergens after intradermal challenge, than healthy horses. In the present study, most of the owners regarded the bites of insects as the main aggravating factor, as has been found earlier [6,7]. However, 24% of the owners mentioned several simultaneous aggravating factors.

In Finland, equine summer eczema affects typically native Scandinavian horse breeds, the Finnhorse and Icelandic horse, and the severity of clinical signs cannot be related to the age at onset nor to the duration of the disease. Equine summer eczema seems to be a multifactoral disease and, therefore, the influence of different environmental agents combined with the individual susceptibility need to be studied further. Summer eczema is one of the equine diseases that most commonly impair the quality of life of horses and cause great financial loss to the owners. Hence, equine summer eczema has to be taken seriously and all efforts to advance research and treatment are to be welcomed.

## Competing interests

The author declares that they have no competing interests.
